# Continuous Kidney Replacement Therapy Practices in Pediatric Intensive Care Units Across Europe

**DOI:** 10.1001/jamanetworkopen.2022.46901

**Published:** 2022-12-15

**Authors:** Marco Daverio, Gerard Cortina, Andrew Jones, Zaccaria Ricci, Demet Demirkol, Paulien Raymakers-Janssen, Francois Lion, Cristina Camilo, Vesna Stojanovic, Serge Grazioli, Tomas Zaoral, Katja Masjosthusmann, Inge Vankessel, Akash Deep

**Affiliations:** 1 Pediatric Intensive Care Unit, Department of Woman’s and Child’s Health, University Hospital of Padua, Padua, Italy; 2 Department of Pediatrics, Medical University of Innsbruck, Innsbruck, Austria; 3 Children’s Acute Transport Service, Great Ormond Street Hospital for Children, National Health Service (NHS) Foundation Trust, London, United Kingdom; 4 Pediatric Intensive Care Unit, Meyer Children’s Hospital, Florence, Italy; 5 Pediatric Intensive Care Medicine, Istanbul Faculty of Medicine, Istanbul, Turkey; 6 Department of Pediatric Intensive Care, Wilhelmina Children’s Hospital/University Medical Center Utrecht, Utrecht, the Netherlands; 7 Department of Cardiothoracic Surgery, Centre Hospitalier Universitaire of Martinique, Fort-de-France, Martinique; 8 Pediatric Intensive Care Unit, Pediatric Department, Hospital de Santa Maria–North Lisbon University Hospital Center, Lisbon, Portugal; 9 Institute for Child and Youth Health Care of Vojvodina Medical Faculty, University of Novi Sad, Novi Sad, Serbia; 10 Division of Neonatal and Pediatric Intensive Care, Department of Pediatrics, Gynecology and Obstetrics, Children’s Hospital, Geneva University Hospitals and Faculty of Medicine, Geneva, Switzerland; 11 Pediatric Intensive Care Unit, Department of Pediatrics, University Hospital of Ostrava, Faculty of Medicine Ostrava, Ostrava, Czech Republic; 12 Department of General Pediatrics, University Children’s Hospital Muenster, Muenster, Germany; 13 Department of Pediatric Intensive Care, Wilhelmina Children’s Hospital, University Medical Center, Utrecht, Utrecht, the Netherlands; 14 Paediatric Intensive Care Unit, King’s College Hospital, NHS Foundation Trust, Denmark Hill, London, United Kingdom; 15 Department of Women and Children’s Health, School of Life Course Sciences, King’s College London, London, United Kingdom

## Abstract

**Importance:**

Continuous kidney replacement therapy (CKRT) is the preferred method of kidney support for children with critical illness in pediatric intensive care units (PICUs). However, there are no data on the current CKRT management practices in European PICUs.

**Objective:**

To describe current CKRT practices across European PICUs.

**Design, Setting, and Participants:**

This cross-sectional survey of PICUs in 20 European countries was conducted by the Critical Care Nephrology Section of the European Society of Pediatric and Neonatal Intensive Care from April 1, 2020, to May 31, 2022. Participants included intensivists and nurses working in European PICUs. The survey was developed in English and distributed using SurveyMonkey. One response from each PICU that provided CKRT was included in the analysis. Data were analyzed from June 1 to June 30, 2022.

**Main Outcome and Measures:**

Demographic characteristics of European PICUs along with organizational and delivery aspects of CKRT (including prescription, liberation from CKRT, and training and education) were assessed.

**Results:**

Of 283 survey responses received, 161 were included in the analysis (response rate, 76%). The attending PICU consultant (70%) and the PICU team (77%) were mainly responsible for CKRT prescription, whereas the PICU nurses were responsible for circuit setup (49%) and bedside machine running (67%). Sixty-one percent of permanent nurses received training to use CKRT, with no need for certification or recertification in 36% of PICUs. Continuous venovenous hemodiafiltration was the preferred dialytic modality (51%). Circuit priming was performed with normal saline (67%) and blood priming in children weighing less than 10 kg (56%). Median (IQR) CKRT dose was 35 (30-50) mL/kg/h in neonates and 30 (30-40) mL/kg/h in children aged 1 month to 18 years. Forty-one percent of PICUs used regional unfractionated heparin infusion, whereas 35% used citrate-based regional anticoagulation. Filters were changed for filter clotting (53%) and increased transmembrane pressure (47%). For routine circuit changes, 72 hours was the cutoff in 62% of PICUs. Some PICUs (34%) monitored fluid removal goals every 4 hours, with variation from 12 hours (17%) to 24 hours (13%). Fluid removal goals ranged from 1 to 3 mL/kg/h. Liberation from CKRT was performed with a diuretic bolus followed by an infusion (32%) or a diuretic bolus alone (19%).

**Conclusions and Relevance:**

This survey study found a wide variation in current CKRT practice, including organizational aspects, education and training, prescription, and liberation from CKRT, in European PICUs. This finding calls for concerted efforts on the part of the pediatric critical care and nephrology communities to streamline CKRT education and training, research, and guidelines to reduce variation in practice.

## Key Points

**Question:**

What are the variations in practice during continuous kidney replacement therapy (CKRT) practice among pediatric intensive care units (PICUs) across Europe?

**Findings:**

In this survey study of 161 European PICUs, substantial variation in CKRT practice was found across various organizational aspects, including follow-up, prescription (eg, anticoagulation), liberation from CKRT, and training and education of staff.

**Meaning:**

The survey found that CKRT management in European PICUs varied widely, which calls for concerted educational initiatives and consensus recommendations on the practice of CKRT.

## Teaser

This survey study reports the demographic characteristics and treatment-related organizational aspects of pediatric intensive care units that deliver continuous kidney replacement therapy across Europe.

## Introduction

Continuous kidney replacement therapy (CKRT) is the preferred method of kidney support in the pediatric intensive care unit (PICU) because it allows for slow fluid removal and solute normalization in children with hemodynamic instability.^1,2,3,4^ Continuous kidney replacement therapy is increasingly being used in the PICU both for kidney and nonkidney indications (acute kidney injury [AKI], fluid overload [FO], electrolyte instability, acid-base imbalances, sepsis [for removal of inflammatory mediators], acute liver failure, inborn errors of metabolism, or drug/toxin removal).^5,6,7,8,9,10^ However, due to the lack of evidence and standardized, practice-based recommendations, wide variations in practice exist in all aspects of delivery of CKRT in children with critical illness.^11,12,13,14,15,16^ Most protocols for CKRT are therefore based on institutional, personal, or historical experience. Although a limited number of pediatric studies^2,3,5,6,7,8,9^ have described the demographic characteristics and outcome data of children with critical illness requiring CKRT, detailed information on current practices for the management of pediatric CKRT in Europe is lacking.

To address this gap, the Critical Care Nephrology Section of the European Society of Paediatric and Neonatal Intensive Care (ESPNIC) conducted a survey with the aim of describing the current CKRT practices across European PICUs. The results of this study may serve as a catalyst for providing training and education in this field to safely deliver CKRT to children with critical illness.

## Methods

### Study Design and Ethics Approval

The Critical Care Nephrology Section of ESPNIC was launched in 2013 and consists of nursing, medical, and allied health professional members. From April 1, 2020, to May 31, 2022, we conducted a cross-sectional anonymous online survey focused on different aspects of CKRT practices in European PICUs. We followed the American Association for Public Opinion Research (AAPOR) reporting guideline.^17^ Since no demographic, observational, or interventional data were collected on patients in this survey study, ethical approval was not deemed necessary in accordance with the Common Rule; however, the project was registered as a quality improvement project with King’s College Hospital in London, UK. Informed consent from participants was implied from their completion of the survey.

### Survey Development and Testing

The survey was developed in English using the online SurveyMonkey (Momentive Inc) instrument for distribution. The survey was designed to address different aspects of CKRT delivery, which we developed based on an extensive review of the literature and expert consensus. This literature review explored important research on the most controversial aspects of CKRT, such as timing of initiation, choice of vascular access, dose of CKRT, anticoagulation, and liberation from CKRT, among others. We searched all available pediatric literature, including single-center studies (mostly retrospective) as well as studies from the multicenter Prospective Pediatric Continuous Renal Replacement Therapy Registry. However, we explored the literature from adult studies when suitable given that most randomized clinical trials have been conducted in adult patients.

Subsequently, the survey was initially pilot-tested among the authors of this study for clarity and face validity. The survey consisted of 78 questions, was divided into different sections, and required about 25 minutes on average to be completed. Detailed information on the data collected is available in the eMethods in Supplement 1, and the survey is provided in the eAppendix in Supplement 1.

### Recruitment of European PICUs and Data Collection

This survey focused on intensivists and nurses working in European PICUs. By using dedicated organizational newsletters, country leads, and personal networks and contacting the ESPNIC Critical Care Nephrology Section members, the questionnaire was spread across 20 countries in Europe. Demographic characteristics of European PICUs along with organizational and delivery aspects of CKRT (including prescription, liberation from CKRT, and training and education) were assessed. No identifiable data regarding staff or patients were collected. We targeted CKRT lead professionals from all units to avoid misdiagnosis of variation in practice based on the level of experience of health care professionals. Responses to the survey were excluded if any of the following conditions were met: the PICU did not perform CKRT, the unit was a neonatal intensive care unit only, and the PICU was outside Europe. We also excluded incomplete responses from the final number of included questionnaires, and, in the case of multiple responses per center, we contacted the CKRT lead of the unit to confirm the entry to be included.

### Statistical Analysis

Raw data that were downloaded from SurveyMonkey were checked for data completeness and potential responses meeting the exclusion criteria. Data were analyzed using Stata, version 17.0 (StataCorp LLC) from June 1 to June 30, 2022. Descriptive data were reported as number and frequency (proportion) for categorical variables and as median (IQR) for continuous variables given their nonparametric distribution.

## Results

### Survey Respondents and PICU Characteristics

The questionnaire received 283 responses. After exclusion criteria were applied (7 PICUs did not perform CKRT, 5 units were neonatal intensive care units, 12 PICUs were outside Europe, and 98 responses were incomplete or duplicates from the same units), 161 responses were included for a response rate of 76% (n = 213) among European PICUs that performed CKRT (Figure). Table 1 summarizes the characteristics of PICUs and respondents as well as organizational aspects of the PICUs involved in the survey. Respondents were mainly physicians (90%) from PICUs with a median (IQR) annual admission rate of 500 (350-800) children.

**Figure.**
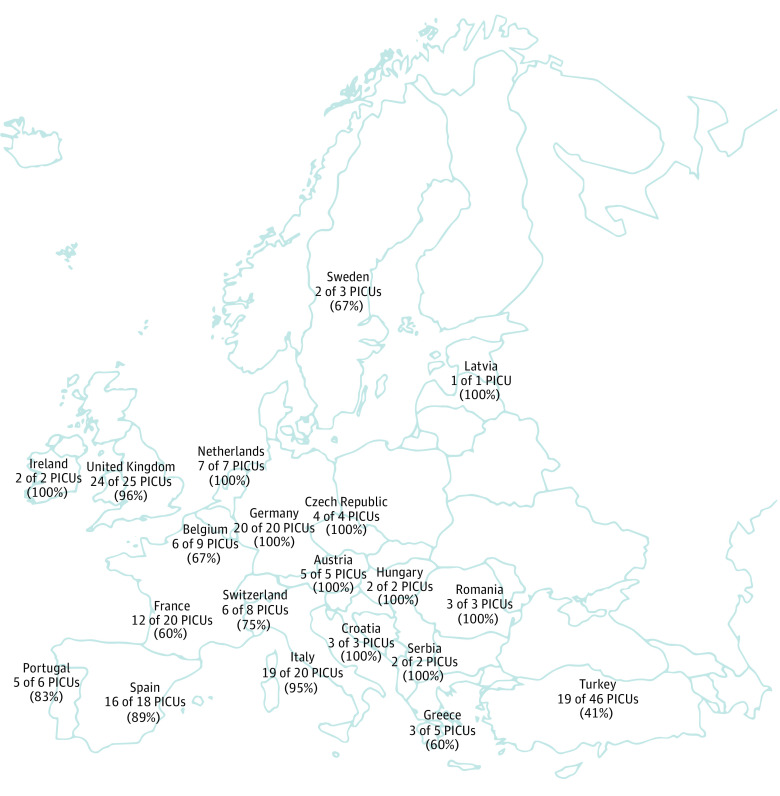
Map Reporting the Distribution of Survey Respondents Across European Countries PICU indicates pediatric intensive care unit.

**Table 1.** Demographic Characteristics and Organizational Aspects of European PICUs Performing CKRT

Characteristic	Responses, No. (%)
Responder profession	
No.	161
Physician	145 (90)
Nurse	16 (10)
PICU dimensions, median (IQR)	
Maximum bed capacity	12 (8-16)
No. of admissions per y	500 (350-800)
Presence of written policy for CKRT	123 (77)
No.	159
Need for and type of consent for CKRT initiation	
No.	141
Verbal consent	61 (43)
Written consent	35 (25)
No consent needed	45 (32)
Minimum weight for starting KRT, median (IQR)	
Peritoneal dialysis	2 (1-3)
CKRT	3 (2-3.5)
Intermittent hemodialysis	6 (5-10)
Person with primary responsibility for prescribing CKRT type or modality to be used^a^	
Attending PICU consultant	113 (70)
Kidney team advice	53 (33)
Unit policy or guidelines	35 (22)
Only 1 KRT modality available	3 (2)
Person with primary responsibility for CKRT management	
No.	141
PICU team	109 (77)
Kidney team	17 (12)
Varies depending on CKRT modality	9 (6)
Combined ICU and kidney team	5 (4)
Technicians	1 (1)
Person with primary responsibility for CKRT setup (lining and priming)	
No.	157
PICU team	
Nurse	77 (49)
Physician	43 (27)
Kidney team	
Nurse	28 (18)
Physician	5 (3)
Perfusionist	4 (3)
Person with primary responsibility for CKRT bedside machine running^a^	
PICU team	
Nurse	107 (67)
Physician	58 (36)
Kidney team	
Nurse	17 (11)
Physician	10 (6)
Perfusionist	2 (1)
Staffing ratio when the patient is both on CKRT and MV	
No.	131
1 Staff member to 1 patient	74 (56)
1 Staff member to 2 patients	32 (24)
2 Staff members to 1 patient	23 (18)
Other ratio	2 (2)
Patients received CKRT follow-up as outpatients by nephrologists	77 (63)
No.	122
Concern regarding the use of adult CKRT machines in children weighing <8 kg	81 (60)
No.	136
Availability of CKRT equipment designed for neonates or infants (ie, CARPEDIEM or NIDUS)	20 (15)
No.	135

Abbreviations: CARPEDIEM, Cardio-Renal Pediatric Dialysis Emergency Machine; CKRT, continuous kidney replacement therapy; ICU, intensive care unit; KRT, kidney replacement therapy; MV, mechanical ventilation; NIDUS, Newcastle Infant Dialysis and Ultrafiltration System; PICU, pediatric intensive care unit.

^a^ The sum of percentages is more than 100% because respondents could indicate more than 1 option.

### Organizational Aspects and CKRT Education

Most PICUs (77%) had a written policy for CKRT management. Initiation of CKRT required written consent in 25% of PICUs. Seventy percent of attending PICU consultants were responsible for prescribing CKRT type or modality. The PICU team (77%) was mainly responsible for CKRT management, whereas in a minority of the cases a nephrologist (12%) managed the treatments. The PICU nurses were mainly responsible for circuit setup (49%) and bedside machine running (67%). Staffing ratios during CKRT varied widely, with 56% of PICUs reporting a ratio of 1 member of staff to 1 patient.

Sixty percent of PICUs expressed concerns regarding the use of adult machines in children weighing less than 8 kg, but only 15% of the PICUs had available dedicated CKRT equipment designed for these children (ie, CARPEDIEM [Cardio-Renal Pediatric Dialysis Emergency Machine; Medtronic] and NIDUS [Newcastle Infant Dialysis and Ultrafiltration System; Allmed Medical Care Holdings Limited]). Sixty-three percent of respondents provided follow-up for patients as outpatients after they were discharged. Sixty-one percent of permanent PICU nurses were formally trained to use CKRT, with no need for certification or recertification in approximately one-third (36%) of PICUs and with need for yearly certification in another one-third (32%) of PICUs. No regular training on CKRT was offered in 59% of PICUs (eTable in Supplement 1).

### Vascular Access Characteristics

eFigure 1 in Supplement 1 describes the current practice regarding location and size for vascular access according to patient age and weight. Ninety-three PICUs used an ultrasonography-guided technique to obtain vascular access to perform CKRT, with the internal jugular vein being the preferred vessel in all age groups (<1 month, 1 month-1 year, 1 year-5 years, and >5 years; preferred by 76%-88%). The size of vascular access increased proportionately with patient weight.

### CKRT Initiation and Termination

Table 2 as well as eFigures 2 and 3 in Supplement 1 show various components of the CKRT prescription. Continuous venovenous hemodiafiltration (CVVHDF) was the most commonly used modality (66%), whereas intermittent hemodialysis (IHD) was the least commonly used (16%) (eFigure 2 in Supplement 1), with 47% of PICUs reporting never using IHD in the past 12 months. Eighty percent of the PICUs offered the possibility to perform tandem therapies, mainly CKRT connected to extracorporeal membrane oxygenation (56%) and CKRT with therapeutic plasma exchange (52%) (eFigure 2 in Supplement 1).

**Table 2.** CKRT Prescription Characteristics

Characteristic	Total responses, No. (%)
Clinical scenario in which the physician would commence CKRT^a^	
No.	91
Severe hyperkalemia	82 (90)
Treating fluid overload in a child with critical illness	77 (85)
Hyperammonemia	77 (85)
Persistent refractory metabolic acidosis	54 (59)
Removing soluble mediators of septic shock	18 (20)
Preventing fluid overload in a child with critical illness	16 (18)
Creating space for nutrition by allowing liberalization of intake	10 (11)
Preferred KRT modality for hyperammonemia	
No.	123
CVVHDF	63 (51)
CVVHD	38 (31)
CVVH	14 (11)
IHD	7 (6)
PD	1 (1)
Dialysate or effluent dose, median (IQR), mL/kg/h	
In neonates	35 (30-50)
In children	30 (30-40)
Blood flow rate, median (IQR), mL/min	
Minimum	20 (10-20)
Maximum	200 (150-200)
Solutions used for circuit priming, excluding blood^a^	
No.	126
Normal saline	84 (67)
Human albumin solution	59 (47)
Plasmalyte	17 (14)
Hartmann solution	8 (6)
Fresh frozen plasma	7 (6)
Indications for blood priming of the circuit^a^	
No.	121
Weight <10 kg	68 (56)
Hemodynamic instability or inotropic requirement	57 (47)
Extracorporeal volume >10% of circulating blood volume	48 (40)
Anemia	45 (37)
Never used	19 (16)
Blood priming performed in all patients receiving CKRT	2 (2)
Modality of blood priming	
No.	128
Priming the whole CKRT circuit with blood	50 (39)
Combining blood and crystalloids for a normal hematocrit	38 (30)
Administering a blood transfusion before starting CKRT	21 (16)
Priming the whole CKRT circuit with blood, or administering a blood transfusion	4 (3)
Combining blood and colloids for a normal hematocrit	2 (2)
Other^b^	1 (1)
Never performed	12 (10)
Preferred dialysate solution	
No.	147
Bicarbonate base solution	107 (73)
Lactate base solution	40 (27)
Customization of the dialysate bag (eg, adding glucose or sodium)	
No.	137
With customization	69 (50)
Position of the replacement fluid	
No.	130
Combination of predilution and postdilution	46 (35)
Postdilution	29 (22)
Predilution	29 (22)
Variable practice depending on patient scenario	26 (20)
Filtration-to-dialysis ratio during CVVHDF	
No.	111
50/50	45 (41)
30/70	35 (32)
Dependent on molecular weight of the target solute	31 (28)
Diuretics (eg, furosemide) during CKRT	
No.	126
Used diuretics	49 (39)
Nutrition support changes during CKRT^a^	
No.	130
Change in protein	62 (48)
Change in calories	49 (38)
Change in trace elements	40 (31)
No changes	52 (40)

Abbreviation: CKRT, continuous kidney replacement therapy; CVVH, continuous venovenous hemofiltration; CVVHD, continuous venovenous hemodialysis; CVVHDF, continuous venovenous hemodiafiltration; IHD, intermittent hemodialysis; PD, peritoneal dialysis.

^a^ The sum of percentages is more than 100% because respondents could indicate more than 1 option.

^b^ Priming the whole circuit first with crystalloid and then blood prime.

eFigure 3 in Supplement 1 shows the modality of choice according to the underlying disease. Continuous venovenous hemodiafiltration was the preferred CKRT modality for all indications except chronic kidney failure, in which IHD and peritoneal dialysis were preferred (eFigure 3A in Supplement 1). The clinical scenarios that prompted initiation of CKRT included severe hyperkalemia (90%), FO (85%), and hyperammonemia (85%). There was wide variation in preference for priming solutions, with normal saline (67%) being most preferred and blood priming being mainly used in children weighing less than 10 kg (56%) and children with hemodynamic instability receiving vasoactive agents (47%) (Table 2). Blood priming was performed by different techniques, including priming the whole CKRT circuit with blood (39%) or combining blood and crystalloids aiming for a normal hematocrit (30%). A bicarbonate base solution (73%) was the preferred solution, and 50% of the PICUs claimed to customize the dialysate bag. The replacement fluid was mainly (35%) administered as a combination of predilution and postdilution, with a filtration to dialysis ratio of 50/50 (41%). More than one-third (39%) of the PICUs used diuretics while the patient was receiving CKRT, and more than half (60%) of the PICUs altered the prescription of nutritional support mainly by changing the amount of protein (48%) and calories (38%) (Table 2).

In case of insufficient desired toxin clearance, respondents preferred to change the CKRT modality, followed by increasing the effluent rate (eFigure 3B in Supplement 1). The CKRT dose was calculated mainly (72%) using a weight-based formula (mL/kg/h), and the median (IQR) CKRT dose among the PICUs was 35 (30-50) mL/kg/h in neonates and 30 (30-40) mL/kg/h in children aged 1 month to 18 years. Only 30% of PICUs reported ever using a CKRT dose over 40 mL/kg/h, with a median (IQR) dose of 80 (45-120) mL/kg/h. The reported median (IQR) minimum blood flow rate was 20 (10-20) mL/min, with a median (IQR) maximum blood flow rate of 200 (150-200) mL/min.

### Anticoagulation Management

Detailed description of anticoagulation strategies is reported in Table 3. First-line anticoagulants of choice were unfractionated heparin (41%) and regional citrate anticoagulation (RCA) (35%). In case of heparin use, activated clotting time (53%) and activated partial thromboplastin time (51%) were the 2 tests used for monitoring. Filters were routinely changed every 72 hours (62%), and 48 to 72 hours were considered an adequate filter life for the majority of PICUs (62%). Filters were changed due to filter clotting (53%) and an increase of transmembrane pressure (47%).

**Table 3.** Anticoagulation Management During CKRT

Characteristic	Responses, No. (%)
First-line anticoagulation of choice	
No.	133
Regional unfractionated heparin infusion	54 (41)
Citrate-based regional anticoagulation	47 (35)
Regional heparin and protamine anticoagulation	15 (11)
Systemic unfractionated heparin infusion	10 (8)
Systemic LMWH	5 (4)
Regional prostacyclin	1 (1)
Other^b^	1 (1)
Second-line anticoagulation of choice	
No.	131
Citrate-based regional anticoagulation	45 (34)
Regional unfractionated heparin	28 (21)
Systemic unfractionated heparin infusion	17 (13)
No anticoagulation	10 (8)
Systemic LMWH	10 (8)
Regional heparin and protamine anticoagulation	9 (7)
Regional prostacyclin	9 (7)
Systemic prostacyclin	1 (1)
Other^c^	1 (1)
Preferred test to measure heparin anticoagulation^a^	
ACT	84 (53)
aPTT	82 (51)
Anti–Xa levels	38 (24)
No monitoring	2 (1)
What is considered an adequate filter life in patients undergoing CKRT?	
No.	132
72 h	19 (14)
48-72 h	82 (62)
24-48 h	25 (19)
≤24 h	6 (5)
When is the filter routinely changed (according to manufacturer recommendations) if there are no issues?	
No.	130
Every 72 h	80 (62)
Every 48 h	13 (10)
Every 24 h	8 (6)
We do not follow manufacturer recommendations	29 (22)
What situation triggers the change of the filter?	
No.	132
Filter clotting	70 (53)
Increase of transmembrane pressure	62 (47)
Most common complications using anticoagulation during CKRT	
No.	91
Catheter and/or circuit thrombosis	27 (30)
Bleeding	22 (24)
Catheter site and/or mucosal bleeding	15 (17)
Citrate overload	12 (13)
Thrombocytopenia	9 (10)
Difficulty achieving adequate anticoagulation	6 (7)

Abbreviations: ACT, activated clotting time; aPTT, activated partial thromboplastin time; CKRT, continuous kidney replacement therapy; KRT, kidney replacement therapy; LMWH, low-molecular-weight heparin.

^a^ The sum of percentages is more than 100% because respondents could indicate more than one option.

^b^ Regional heparin without protamine.

^c^ Direct thrombin inhibitors or no answer.

### Fluid Removal Strategies and Liberation From CKRT

Regarding fluid removal during CKRT, 7% of PICUs combined both fluid balance and hemodynamic status in their decision to initiate fluid removal during CKRT. Thirty-two percent of PICUs set a maximum net ultrafiltration rate, ranging from 1 to 3 mL/kg/h. The achievement of the desired fluid removal goals was assessed every 4 hours (34%), with some PICUs assessing this factor on an ad hoc basis, varying from 12 hours (17%) to 24 hours (13%).

Regarding liberation from CKRT, native urine output and the resolution of FO were the 2 factors most often associated with the decision to perform a trial of liberation from CKRT (eFigure 4A in Supplement 1). Liberation from CKRT was performed mainly with a diuretic bolus followed by an infusion (32%) or by a diuretic bolus alone (19%), and 34% of PICUs used a variable strategy depending on the patient’s clinical characteristics (eFigure 4B in Supplement 1). Nine percent of PICUs did not use any agent (ie, fluid and/or diuretic) to trial a patient off of CKRT.

## Discussion

Although a few studies have explored practices and attitudes toward the management of CKRT in adult patients in the ICU, to our knowledge, this cross-sectional survey was the first to explore the current status of CKRT management in European PICUs.^18,19^ The response rate among PICUs performing CKRT was high (76%), and therefore the results can be considered representative of current CKRT practice across European PICUs. We found wide variation in current CKRT management, reinforcing the need for consensus on best practice guidelines.

Continuous kidney replacement therapy is a complex extracorporeal therapy requiring a high level of expertise, training, and resources.^1,20^ Given that children with critical illness receive CKRT in the PICU, it is not surprising that, in this study, the multidisciplinary PICU team was primarily in charge of CKRT in most units and, in a few units, CKRT was primarily managed by nephrologists. Each strategy has advantages and disadvantages, with a collaborative approach being the best strategy.

We found low rates of education and training being offered to health care professionals who perform CKRT. It has been shown that troubleshooting skills and adverse incidents are directly associated with regular training of staff involved in providing CKRT.^21,22^ This outcome is an important finding on which the ESPNIC Critical Care Nephrology Section will base the design of ongoing education and training in this field.

Most PICUs offer a range of CKRT modalities (continuous venovenous hemofiltration, continuous venovenous hemodialysis, and CVVHDF), perhaps reflecting the versatility of modern CKRT machines. The survey results showed CVVHDF to be the most used modality in all proposed clinical scenarios except for chronic kidney failure, in which peritoneal dialysis and IHD were most often used. Theoretically, CVVHDF has the advantage of combining small-molecule elimination by diffusion with medium-sized solutes by convection. Moreover, adding a replacement solution in predilution reduces the risk of clotting and, when combined with RCA, facilitates citrate elimination by diffusion.^23,24^ However, to our knowledge, no study has examined the superiority of 1 modality over the other, and typically each PICU applies the modality they are most comfortable using.

Regarding the indications for initiation of CKRT, respondents indicated that the most common clinical scenarios triggering prompt initiation of CKRT were severe hyperkalemia, FO, and hyperammonemia. Fluid overload with or without AKI is common in children with critical illness,^25,26^ and it has been shown to be associated with increased mortality; therefore, expert consensus recommends the initiation of CKRT when FO reaches the threshold of 10% and response to diuretics is insufficient.^5,6,7,27^ More than one-third of PICUs used diuretics during CKRT treatment, although the Kidney Disease: Improving Global Outcomes (KDIGO) guidelines do not recommend their use to increase urine output or reduce the duration of CKRT.^16^

Sixty percent of respondents expressed concerns regarding the use of adult CKRT machines in children weighing less than 8 kg. A dedicated neonatal or infant dialysis machine, CARPEDIEM, was introduced in clinical practice, while another machine, NIDUS, is currently under investigation.^28,29,30,31^ However, only 15% of PICUs described availability of one of these dedicated neonatal or infant machines. Nonetheless, it should be highlighted that third- and fourth-generation machines have repeatedly been shown to have a high level of accuracy in children, and dedicated technology should be strongly recommended in children weighing less than 8 to 10 kg, especially because of smaller priming volumes and accurate ultrafiltration rates.^32,33^

There appeared to be a consensus that blood priming of the circuit is needed in cases of body weight less than 10 kg, hemodynamic instability, or extracorporeal blood volume exceeding 10% of the patient´s blood volume. However, wide variations existed in the technique to perform blood priming. Since blood priming can be technically challenging, having a common ground for PICUs that practice this technique might be helpful.

We found good adherence to the KDIGO guidelines recommendation that vascular access be placed under ultrasonographic guidance in the internal jugular vein.^16^ The recommendation has traditionally been to insert a large vascular catheter to maximize blood flow, but also to be mindful of vascular thromboembolism if the catheter-to-vessel ratio exceeded 45%.^34,35^

The majority of PICUs used weight-based CKRT doses as opposed to body surface area–based calculations. It seems important to get a consensus in children because dose calculations based on body surface area tend to be higher in neonates and infants (eg, in an infant, a dose of 2 L/1.73 m^2^/h corresponds to approximately 60 mL/kg/h).^36^ We found considerable variation in dose used, with only one-third of PICUs reporting the use of a CKRT dose over 40 mL/kg/h. The role of high-volume CKRT is still uncertain in both pediatric and adult populations.^35,37,38,39^ However, one-third of respondents increased the effluent dose in case of inadequate clearance of toxins. Although large randomized clinical trials performed in adult patients have not shown any survival benefit when high-intensity CKRT was compared with standard-dose CKRT, there is no concrete evidence in pediatrics.^35,37,38^ Surviving sepsis guidelines for children do not recommend high-volume hemofiltration for sepsis.^40^ However, high-volume CKRT might be associated with underdosing of essential drugs (eg, antibiotics and antiepileptics) and an exacerbated catabolic state already present in AKI by removing essential nutrients. As with every other aspect of CKRT, optimal delivery of CKRT should be a dynamic and patient-specific therapy, taking into account the potential discrepancy between the prescribed and delivered dose, which could be as high as 20% to 30%.^35^

The 2 most common anticoagulants used were regional unfractionated heparin and RCA. Regional citrate anticoagulation has been recommended by KDIGO guidelines as the first choice in patients who do not have a contraindication for citrate because RCA is strictly an extracorporeal anticoagulant and reduces bleeding complications.^16^ Several prospective randomized clinical trials^41,42^ in adult patients have demonstrated increased circuit life in CKRT with RCA. Acceptable filter life as well as the safety and efficacy of RCA have also been demonstrated in case series of children aged 1 month to 10 years.^43,44,45,46,47^ Although the largest study by the Prospective Pediatric Continuous Renal Replacement Therapy Registry^45^ showed no difference in circuit life between these 2 anticoagulation methods, RCA was associated with fewer bleeding complications. A recent systematic review^48^ seemed to highlight the improved safety and efficacy profile of RCA compared with systemic heparin anticoagulation in the pediatric population. In rare cases where both RCA and systemic heparin anticoagulation are contraindicated, such as in acute liver failure, prostacyclin or nafamostat mesylate have been shown to be promising alternatives^49^; however, in the present survey, these agents were rarely used.

Timing, amount of fluid removal, and fluid removal goals are all important practical issues encountered by health care professionals. The aim of fluid removal during CKRT is to balance the desired clinical goals (eg, oxygenation and control of FO) with avoiding the adverse effects of overzealous fluid removal (eg, hypotension, acid-base disturbances, and end-organ ischemia).^50,51,52^ The best approach for initial and subsequent fluid removal rates in neonates and children is dynamic and in accordance with the physiological and hemodynamic status of the patient. In this survey study, different PICUs set hourly, 4- to 24-hour, or ad hoc fluid assessment goals. Achievement of the net ultrafiltration goal is an important aspect of the CKRT prescription that is often overlooked, leading to suboptimal achievement of fluid balance goals. Based on hemodynamic status, 32% of PICUs set a maximum net ultrafiltration rate: the actual outcome of the interaction between cumulative FO and maximum net ultrafiltration remains to be clarified.

The KDIGO guidelines recommend cessation of CKRT when it is no longer needed because native kidney function has adequately recovered and/or in accordance with the patient´s goals of care. In this survey study, we found that current practice for deciding the timing of liberation from CKRT varied widely. Some clinicians used native urine output during CKRT (variable amounts), whereas others evaluated clinical improvement and/or resolution of FO, with some studies suggesting that biomarkers could be used to estimate successful liberation from CKRT. A recent systematic review and meta-analysis^53^ in adults showed 16 variables associated with successful CKRT discontinuation. Of these variables, urine output seemed to be the most reliable.^53^ In cases of unsuccessful liberation from CKRT, timing and choice of transition to other therapeutic modalities remain unclear. Similarly, there remains a wide variation in the practice adopted by clinicians to trial patients off CKRT, as found in this study.

### Strengths and Limitations

To our knowledge, this survey study is one of the largest studies to explore CKRT practices in European PICUs. Getting responses from CKRT lead professionals at each PICU meant that we could potentially avoid misdiagnosing variation in practice based on the level of experience of health care professionals. However, the study had some limitations. First, we could not retrieve data from each European country partly due to lack of contacts, knowledge about the respective infrastructure, and language barriers. Nevertheless, we had a response rate of 76%; therefore, we believe the findings provide a fair representation of CKRT practices across Europe. Second, we could not explore all aspects of CKRT due to how extensive the survey would have become. Third, since the survey was limited to European PICUs, we could not extrapolate the findings to PICUs in other regions where CKRT may be provided by varying health care professionals or where resources may differ. Fourth, the possibility of respondent bias could not be avoided, being inherent to every survey study.

## Conclusions 

We found wide variations in practice even among experienced clinicians offering CKRT in European PICUs. This finding calls for dedicated pediatric critical care nephrology consortia, such as the Acute Disease Quality Initiative and the Critical Care Nephrology Section of ESPNIC, to engage in global initiatives to streamline education and training, research, and guidelines that can be easily accessed by stakeholders in the field to reduce variation in practice.

## Notes

Supplement 1. **eMethods.** Data Collected With the Survey **eTable.** Nursing Staff Education and Training for CKRT Among the PICUs **eFigure 1.** Vascular Access Location and Size Preferences According to Age and Weight **eFigure 2.** KRT Modalities Most Commonly Used in the Previous 12 Months in the PICUs (eFigure 2A) and Tandem Therapies Available (eFigure 2B) **eFigure 3.** KRT Modality According to the Disease (eFigure 3A) and CKRT Strategies in Case of Lack of Toxins Clearance (eFigure 3B) **eFigure 4.** Factors Influencing Decision to Stop CKRT (eFigure 4A) and Strategies for CKRT Discontinuation (eFigure 4B) **eAppendix.** Survey

Supplement 2.
Nonauthor Collaborators

Supplement 3.
Data Sharing Statement
