# Supplementary material for: Continuous Kidney Replacement Therapy Practices in Pediatric Intensive Care Units Across Europe
Source: JAMA Netw Open. 2022 Dec 15;5(12):e2246901. doi: 10.1001/jamanetworkopen.2022.46901 (PMC9856326; doi:10.1001/jamanetworkopen.2022.46901)
Supplement: Supplement 1. — eMethods. Data Collected With the Survey eTable. Nursing Staff Education and Training for CKRT Among the PICUs eFigure 1. Vascular Access Location and Size Preferences According to Age and Weight eFigure 2. KRT Modalities Most Commonly Used in the Previous 12 Months in the PICUs (eFigure 2A) and Tandem Therapies Available (eFigure 2B) eFigure 3. KRT Modality According to the Disease (eFigure 3A) and CKRT Strategies in Case of Lack of Toxins Clearance (eFigure 3B) eFigure 4. Factors Influencing Decision to Stop CKRT (eFigure 4A) and Strategies for CKRT Discontinuation (eFigure 4B) eAppendix. Survey [file jamanetwopen-e2246901-s001.pdf]

Supplemental Online Content

Daverio M, Cortina G, Jones A, et al. Continuous kidney replacement therapy practices in pediatric intensive care units across Europe. *JAMA Netw Open*. 2022;5(12):e2246901. doi:10.1001/jamanetworkopen.2022.46901

eMethods. Data Collected With the Survey

eTable. Nursing Staff Education and Training for CKRT Among the PICUs

eFigure 1. Vascular Access Location and Size Preferences According to Age and Weight

eFigure 2. KRT Modalities Most Commonly Used in the Previous 12 Months in the PICUs (eFigure 2A) and Tandem Therapies Available (eFigure 2B)

eFigure 3. KRT Modality According to the Disease (eFigure 3A) and CKRT Strategies in Case of Lack of Toxins Clearance (eFigure 3B)

eFigure 4. Factors Influencing Decision to Stop CKRT (eFigure 4A) and Strategies for CKRT Discontinuation (eFigure 4B)

eAppendix. Survey

This supplemental material has been provided by the authors to give readers additional information about their work.

© 2022 Daverio M et al. *JAMA Network Open.*

eMethods. Data Collected With the Survey

The following data were collected in the survey: i) PICU demographic aspects including: PICU and patients characteristics, PICU location, and type and size of the PICU; ii) CKRT organizational aspects including: information on CKRT modalities used, the availability of tandem therapies, the use of a CKRT written policy, the professionals responsible for the CKRT provision and bedside management, the staffing ratio for patients on CKRT, information regarding CKRT training and education, and availability of CKRT equipment designed for small children (< 8 kg); iii) information regarding CKRT prescription, including: vascular access (preferred site and size according to the age/weight) and its management while the CKRT is not in use, technical aspects such as the minimum weight for the CKRT, CKRT dose, dialysate/effluent doses, blood flow rates, solutions used both for priming and as dialysis/replacement fluid, use of pre or post-dilution replacement fluid, timing, amount and assessment of fluid removal for fluid overload, and timing and techniques of liberation from CKRT, details regarding the anticoagulation choice, monitoring and side-effects, and change of nutritional prescription during CKRT; iv) prescription of CKRT for common PICU conditions like hyperammonaemia, liver failure and septic shock. We used single- and multiple-choice questions, closed-ended and free text questions to allow for comprehensive detailed information on each topic and to facilitate data analyses and comparisons.

© 2022 Daverio M et al. *JAMA Network Open.*

**eTable.** Nursing staff education and training for CKRT among the PICUs.	
**Characteristics**	**Responses, No. (%)**
Which nursing staff is trained to use CKRT [n=122]	
- All permanent PICU nurses	75 (61)
- Only selected staff	47 (39)
How often the nursing need to be re-certified for CKRT in the unit [n=122]	
- No certification/recertification needed	44 (36)
- Yearly	39 (32)
- 2 yearly	26 (21)
- Irregularly offered	9 (7)
- 3 yearly	3 (3)
- 5 yearly	1 (1)
How often the nursing receive CKRT training in the unit [n=125]	
- No regular courses offered	74 (59)
- Yearly requirement	41 (33)
- 6 monthly	5 (4)
- More regularly than 6 monthly	5 (4)
CKRT: continuous kidney replacement therapy	

© 2022 Daverio M et al. *JAMA Network Open.*

eFigure1: Vascular access location and size preferences according to age and weight

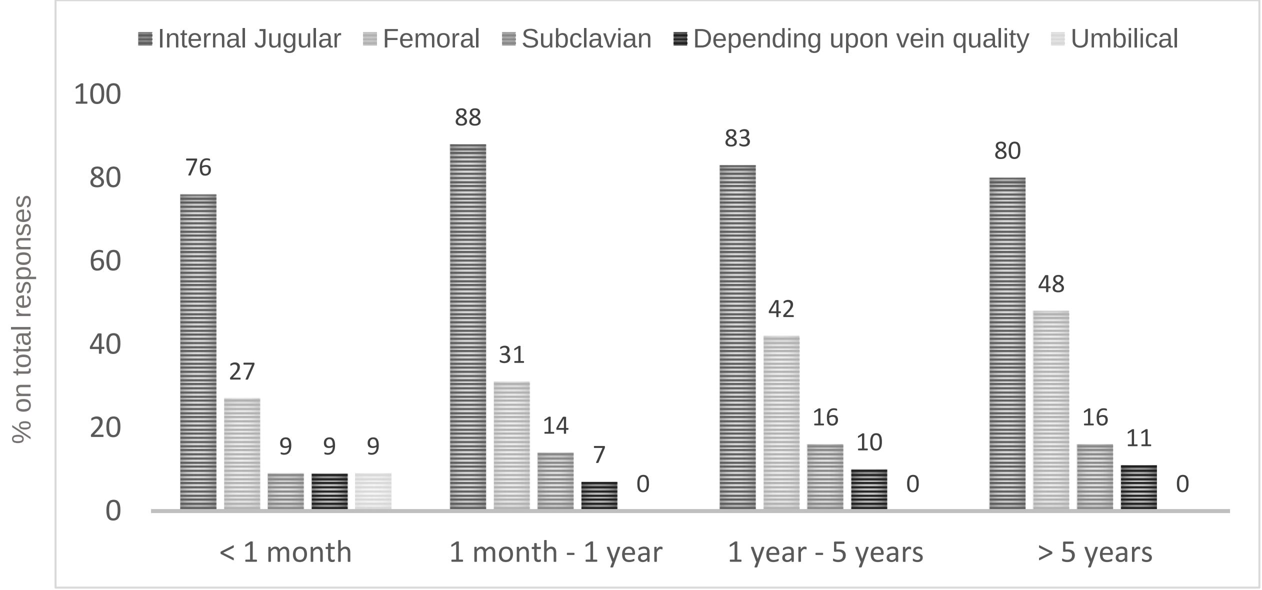

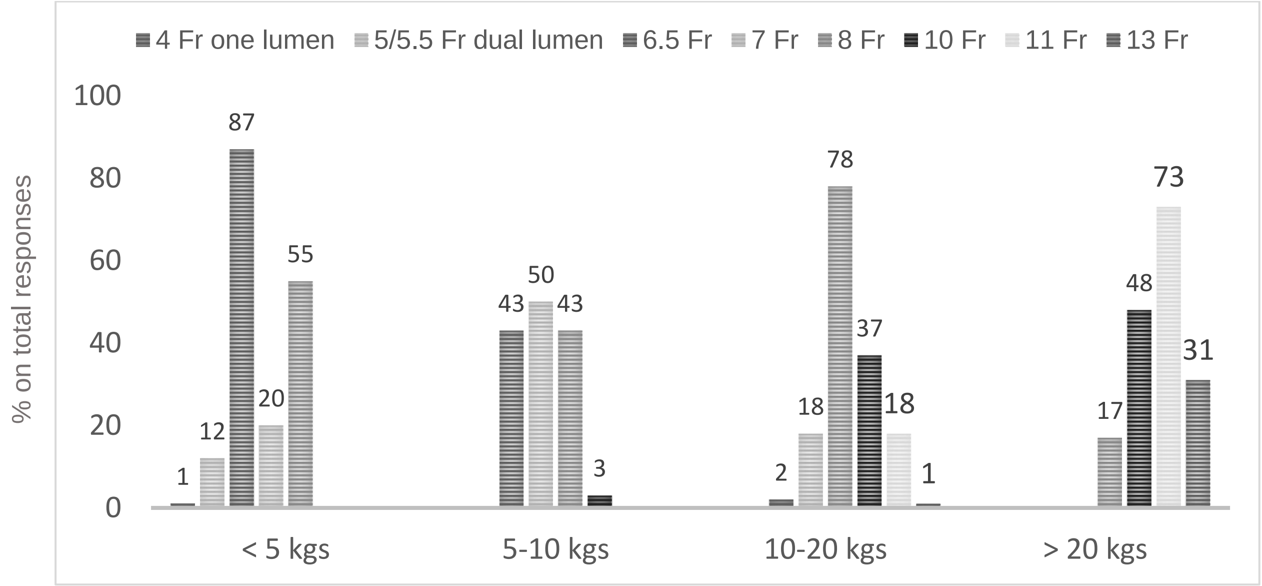

© 2022 Daverio M et al. *JAMA Network Open.*

eFigure 2: KRT modalities most commonly used in the previous 12 months in the PICUs (eFigure 2A) and tandem therapies available (eFigure 2B).

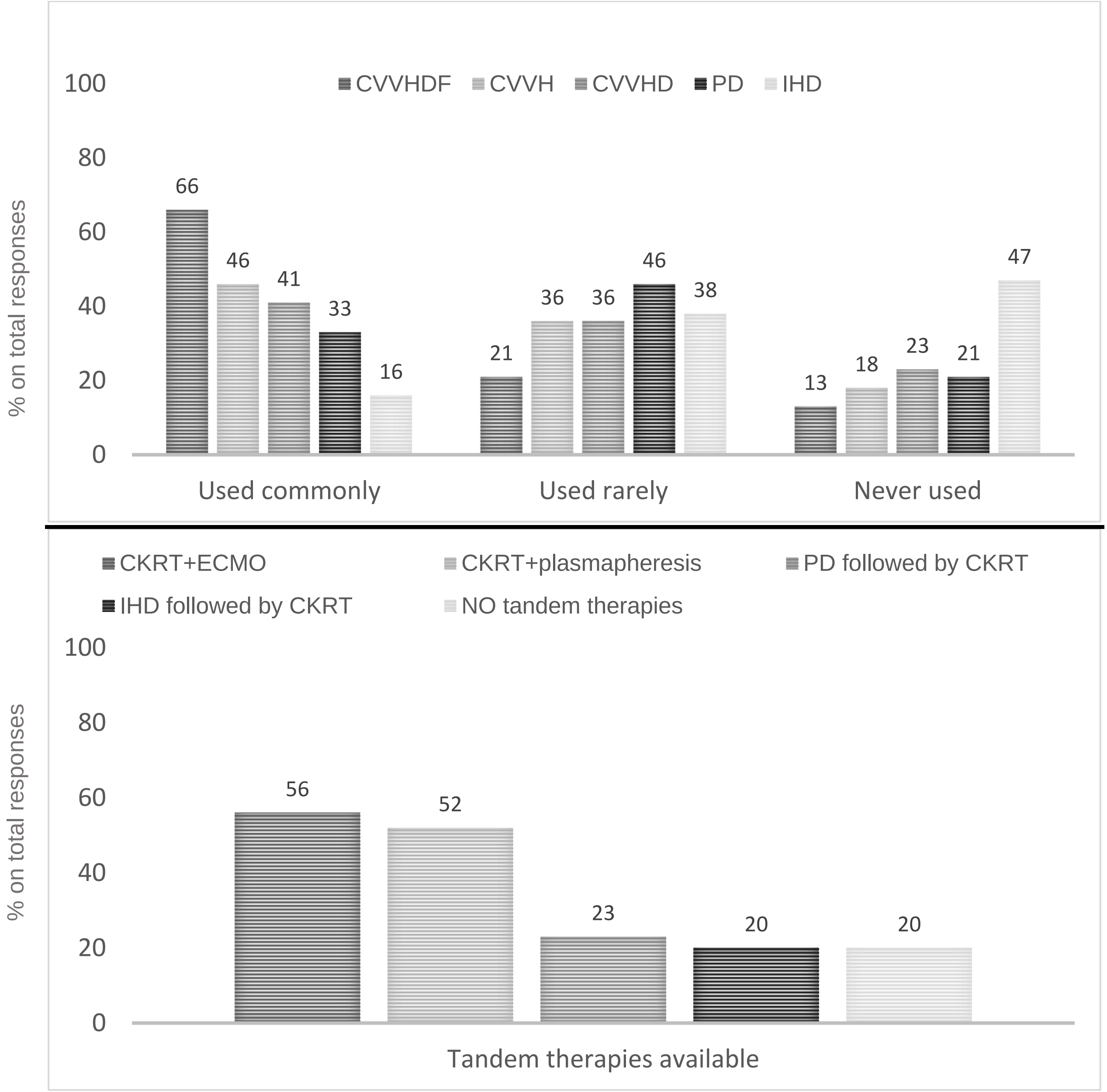

© 2022 Daverio M et al. *JAMA Network Open.*

eFigure 3: KRT modality according to the disease (eFigure 3A) and CKRT strategies in case of lack of toxins clearance (eFigure 3B).

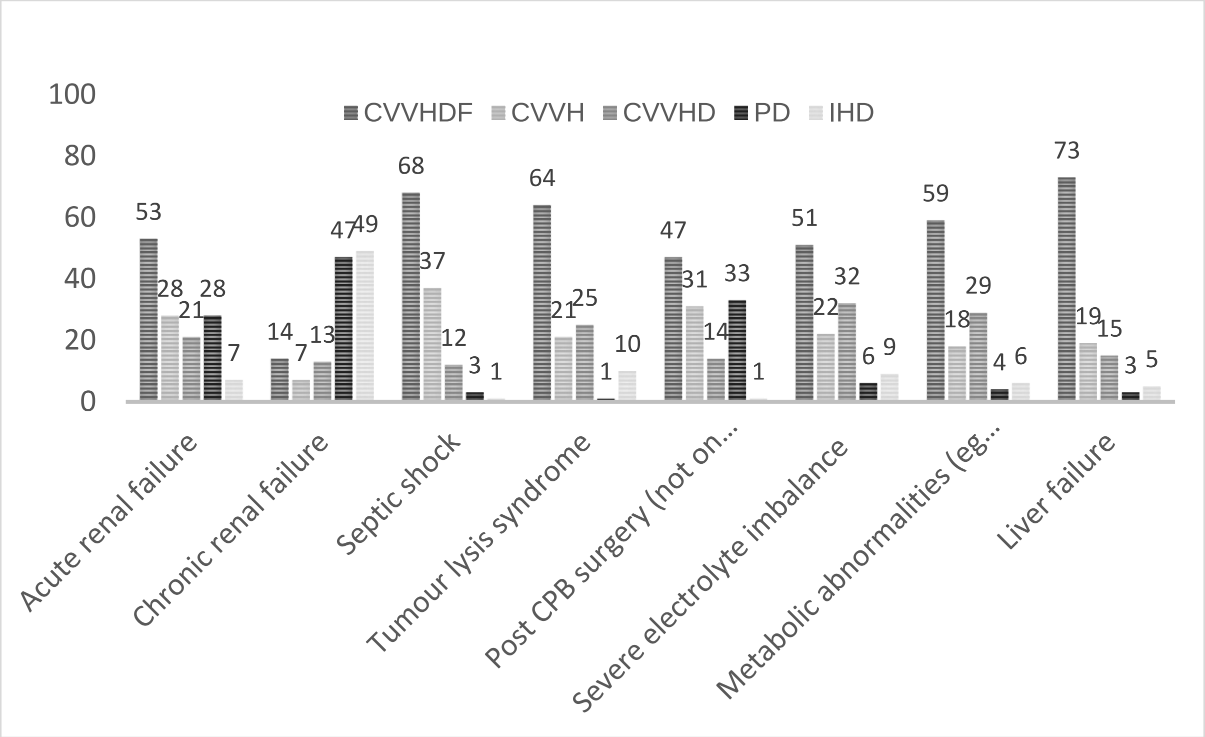

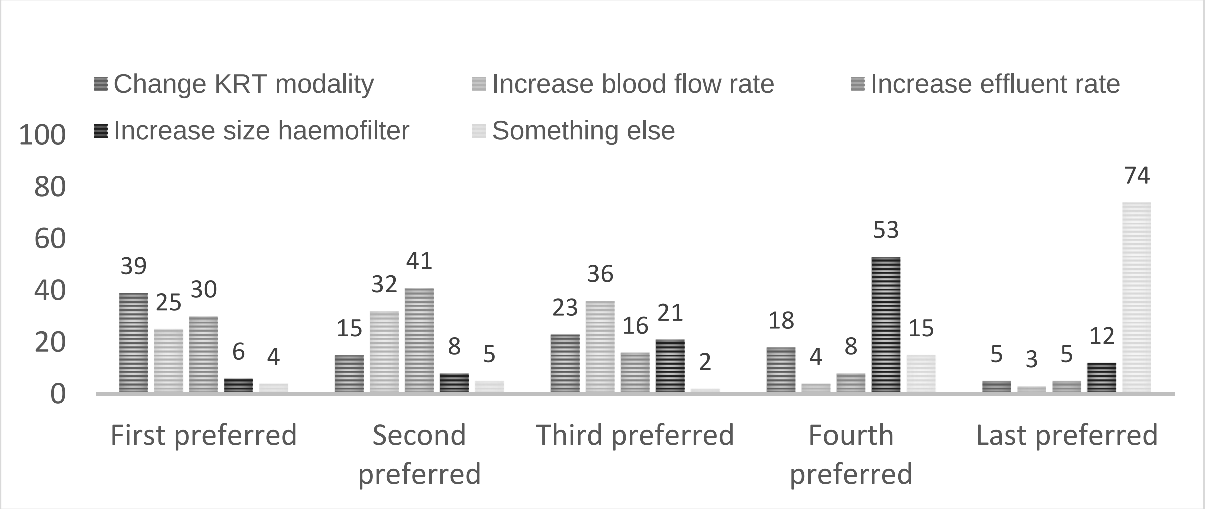

© 2022 Daverio M et al. *JAMA Network Open.*

eFigure 4: Factors influencing decision to stop CKRT (eFigure 4A) and strategies for CKRT discontinuation (eFigure 4B).

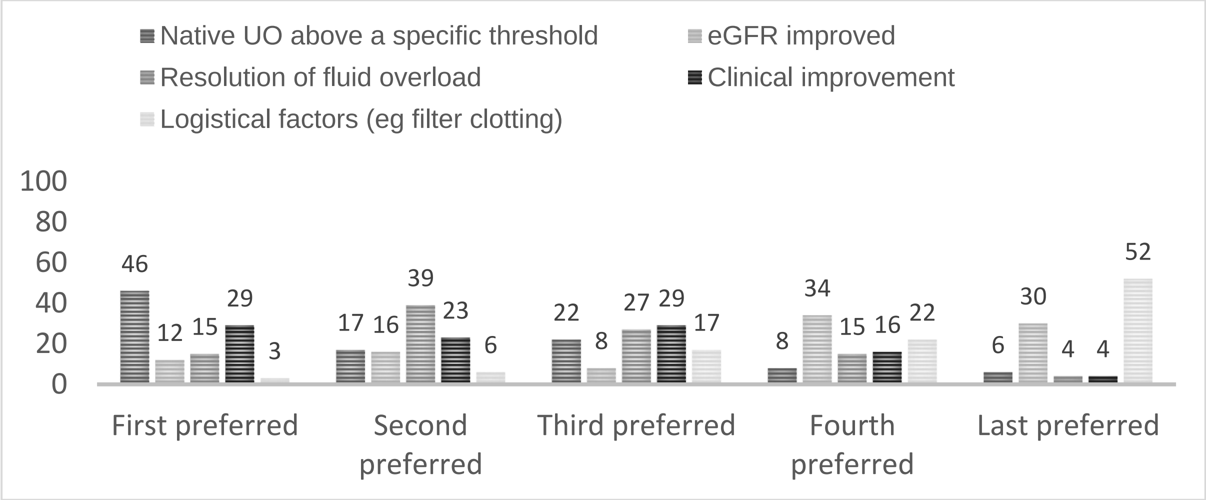

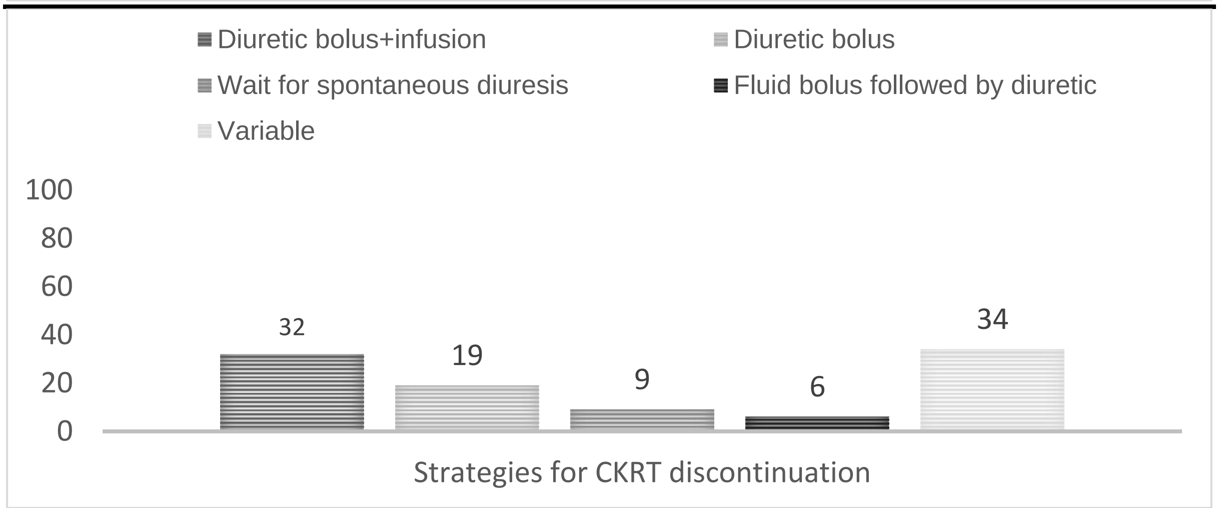

© 2022 Daverio M et al. *JAMA Network Open.*

eAppendix. Survey

INTRODUCTION

We are carrying out a survey regarding Continuous Renal Replacement Therapy (CRRT) practice across Europe

As you are aware RRT is practised differently across the PICUs, with different levels of expertise and experience.

The survey result will be used for understanding the current RRT practices across UK and Europe PICUs and the training available.

We will be also interested in getting a consensus practice statement, if possible, and review of the paediatric RRT guidelines where needed.

The results of the survey will be widely shared as collective data and individual unit responses will remain anonymous and confidential.

Where necessary, please do approach other members of your unit to gather information for questionnaire completion.

The questionnaire will take a total of approximately 15-20 minutes to complete. We are very grateful for your efforts in the completion of this survey.

Regards
Akash Deep and Vesna Stojanovic on behalf
ofRenal/CRRT section of ESPNIC

Glossary:
RRT (Renal replacement therapy) – any therapy that replaces the normal blood-filtering function of the kidneys. It can be continuous or intermittent.
CRRT – Continuous renal replacement
therapy CVVH – Continuous veno-venous
haemofiltration CVVHD – Continuous veno-
venous haemodialysis
CVVHDF – Continuous veno-venous
haemodiafiltrationIHD – Intermittent haemodiaysis
PD – Peritoneal Dialysis
SLED – Slow low efficiency daily dialysis

UNIT DATA

1. Hospital ______

2. Country ______

3. Are you a…
    - o Doctor
    - o Nurse

4. Your specialty________

© 2022 Daverio M et al. *JAMA Network Open.*

5. Your grade________

6. Are you the unit lead for RRT?

- Yes
- No

7. Number of Pediatric Intensive Care beds ______

8. Paediatric specialities available in your unit:

- Cardiology
- Cardiac Surgery
- Neurology
- Neurosurgery
- Trauma
- ENT
- General Surgery
- Oncology
- Hepatology
- Respiratory
- General Paediatrics
- Nephrology

9. Annual number of admissions (per most recent unit data) ____

10. Which modalities of RRT do you offer? (select all that apply)

- PD
- IHD
- SLED
- CVVH
- CVVHD
- CVVHDF
- No RRT offered

11. How many RRT bed-days do you record in a year? ____

12. Does your unit have a written CRRT policy? ____

- Yes
- No

13. On your unit what is the typical interval (in hours) between the decision to start CRRT and the initiation of CRRT?

MODALITIES OF RRT

14. Modalities of RRT used in the last 12 months on your unit?

	Used commonly	Used rarely	Never used
PD			
CVVH			
CVVHD			
CVVHDF			
Other			

© 2022 Daverio M et al. *JAMA Network Open.*

Other (please specify) _______________

15. Who has primary responsibility for the management of RRT on your unit?

- o The ICU team
- o The renal team
- o Perfusionists
- o It varies depending on modality of RRT

16. If your unit offers more than one modality of RRT, how is the decision made on which modality to use for each patient? (select all that apply)

- o Decision by attending PICU consultant
- o Unit policy / guidelines
- o On renal team advice
- o N/A - only one modality available
- o Other (please specify)

17. What is your preference of RRT modality for treatment of the following conditions?

	PD	CVVH	CVVHD	CVVHDF	IHD	Other
Acute renal Failure						
Chronic renal failure						
Septic Shock						
Tumour lysis syndrome						
Post Cardiopulmonary Bypass surgery (not on ECMO)						
Severe electrolyte imbalance e.g. hyperkalemia						
Metabolic abnormalities e.g. hyperammonaemia						
Liver failure						

18. Does your unit have minimum weight restrictions for the following modalities? (please state value in kg)

PD ________
CRRT ________
IHD ________

19. In which of the following scenarios would you consider commencing **CRRT**? Select as many as apply.

- o Treating fluid overload in a critically ill child Severe
- o Hyperkalaemia
- o Persistent refractory metabolic acidosis
- o Hyperammonemia in inborn errors of metabolism and liver failure
- o Prevention of fluid overload in a critically ill child
- o Removing soluble mediators of septic shock
- o To ‘create space’ for nutrition by allowing liberalisation of intake

20. What dialysate/effluent flow rate you would consider using for a **neonate** receiving CRRT? (in ml/kg/hr)

____

21. What dialysate/effluent flow rate you would consider using for a **child** receiving CRRT? (in ml/kg/hr)

© 2022 Daverio M et al. *JAMA Network Open.*

____

22. What are the minimum and maximum blood flow rates used on your unit? (ml/min)

Minimum ________

Maximum ________

23. How do you consent parents/children for RRT?

- Written consent
- Verbal consent
- Specific consent not considered necessary

VASCULAR ACCESS

24. What is your preferred site of vascular access for CRRT or IHD? (more than one option can be selected)

	Femoral	Internal Jugular	Subclavian	Umbilical	Other	No preference / depending upon veins
Less than 1 month						
1 month to 1 year						
1 to 5 years						
Over 5 years						

25. Please select which size of dual lumen catheter you would consider using for the following weight categories (select all that apply).

	6.5 Fr	7 Fr	8 Fr	10 Fr	11 Fr	13 Fr	Other
Less than 5 kg							
5-10 kg							
10-20 kg							
>20 kg							
Other (please specify) ________							

26. Is it routine in your unit to employ ultrasound guidance for siting of vascular catheters?

- Yes
- No

27. While a dual lumen vascular catheter is not in use, which of the following techniques do you employ to maintain patency?

- Dead space of each lumen filled with heparin solution (priming volume) and clamped off

© 2022 Daverio M et al. *JAMA Network Open.*

- Slow infusion of crystalloid through each lumen
- Flushed with crystalloid and clamped off
- Other (please specify) ________

EQUIPMENT

28. How do you perform PD?

- A PD machine
- Manually

29. What dialysate solutions do you routinely use?

- A commerical lactate base solution
- A commercial bicarbonate based solution
- Other (please specify) ________

30. Do you ever use custom made dialysate solutions, or adjust the content (e.g. adding glucose or sodium) of pre-made bags?

- Yes
- No

31. Do you have concerns about the safety and efficacy of using extracorporeal RRT systems designed for adults in smaller children (<8kg)?

- Yes
- No

32. Does your unit use any specialised RRT equipment designed for smaller children, such as the CARPE DIEM device for CRRT or the NIDUS device for haemodialysis?

- Yes
- No

33. Do you think the use of such miniaturised devices in future will lead to safer and more efficacious extracorporeal RRT for smaller children, compared to the current practice of using adult devices off license?

- Yes
- No

ANTICOAGULATION

34. What is your **first** line choice of anticoagulation for CRRT (assuming the child has normal coagulation status)?

- Citrate based regional anticoagulation
- Regional heparin and protamine anticoagulation
- Regional unfractionated heparin
- Systemic LMWH
- Regional prostacyclin
- No anticoagulation
- Other (please specify)

35. What is your **second** line choice of anticoagulation for CRRT (assuming the child has normal coagulation

© 2022 Daverio M et al. *JAMA Network Open.*

status)?

- Citrate based regional anticoagulation
- Regional heparin and protamine anticoagulation
- Regional unfractionated heparin
- Systemic LMWH
- Regional prostacyclin
- No anticoagulation
- Other (please specify)

36. What is your preferred test(s) for measuring heparin anticoagulation? (select all that apply)

- Activated Clotting Time
- Coagulation profile – APTT
- Anti Xa levels
- No monitoring
- We don't use heparin on our unit
- Other (please specify) _________

37. On your unit, approximately what percentage of anticoagulated patients on CRRT experience clinically important bleeding?

____

38. On your unit, what is the most commonly experienced complication of using anticoagulation on a child recieving CRRT?

____

39. In a coagulopathic patient, do you have a threshold for reducing or discontinuing circuit anticoagulation? Please describe your practice below.

____

40. What would you consider an acceptable filter life in patients treated with CRRT on anticoagulation?

- Up to 24 hours
- 24-48 hours
- 48-72 hours
- > 72 hours

41. At what point would you change the filter?

- When transmembrane pressure is raised
- When the filter clots

RUNNING CRRT

42. Who performs the set up ('lining and priming') of the CRRT system?

- Intensive care nurse
- Renal nurse
- A perfusionist

© 2022 Daverio M et al. *JAMA Network Open.*

- An ICU doctor
- A renal doctor
- Other (please specify)__________

43. Who performs the bedside running of the CRRT system?

- Intensive care nurse
- Renal nurse
- A perfusionist
- An ICU doctor
- A renal doctor
- Other (please specify)__________

44. What are the indications in your unit for priming the CRRT circuit with blood?

- Weight < 10kg
- Extracorporeal volume >10% of circulating blood volume
- Haemodynamic instability / inotropic requirement
- Anaemia
- All patients
- Blood prime never used
- Other (please specify) __________

45. How do you perform a 'blood prime'?

- Administer a blood transfusion before starting CRRT
- Prime the whole CRRT circuit with blood
- A combination of blood and crystalloid aiming for a normal haemocrit
- Other (please specify)

46. When not priming the circuit with blood, what other solutions do you use?

- Sodium Chloride
- Huma Albumin Solution (HAS)
- Harmann's Solution
- Fresh Frozen Plasma (FFP)
- Plasmalyte
- Other (please specify) _________

47. What is the bedside staffing ratio when a child is both on CRRT and requires mechanical ventilation?

- 1 member of staff to 1 patient
- 2 members of staff to 1 patient
- Other (please specify)

48. As per the manufacturer's recommendation, when would you routinely change the haemofilter?

- Every 24 hours
- Every 48 hours
- Every 72 hours
- We do not follow the manufacturers recommendations

49. If you practice CVVH and/or CVVHDF, where do you routinely add the replacement fluid in the circuit?

- Pre-dilution (before blood passes through filter)
- Post-dilution (after blood passes through filter)
- Combination of pre- and post- dilution
- Variable practice depending on the patient scenario

50. When using CVVHDF, what ratio of filtration to dialysis do you use?

- 50/50
- 30/70
- Dependent on molecular weight of the target solute
- CVVHDF not used
- Other (please specify) _________

51. Do you alter the the nutritional prescription once CRRT is commenced?

- Yes
- No

52. If yes, what do you change? (select all that apply)

- Calories
- Protein
- Trace elements

TOXIN CLEARANCE

53. In cases where the toxin is not being cleared at the desired rate, what are your preferred interventions? (rank preferred interventions 1-5)

	1	2	3	4	5
Change the modality of RRT (eg. From CVVH to CVVHDF)					
Increase the blood flow rate					
Increase the effluent rate					
Increase the size of the haemofilter					
Something else					

54. For hyperammonaemia secondary to an inborn error of metabolism, what is your preferred modality of CRRT?

- IHD
- CVVH
- CVVHD
- CVVHDF
- PD

55. Do you perform any of the following tandem therapies? (select all that apply)

- ECMO and CRRT
- CRRT and Plasmapheresis
- IHD followed by CRRT
- PD followed by CRRT
- We do not perform tandem therapies

SEPTIC SHOCK

56. What are your indications for starting CRRT in septic shock? (select as many as apply)

- Cytokine removal

© 2022 Daverio M et al. *JAMA Network Open.*

- Fluid overload
- Oligo-anuria
- To make space for nutrition
- To correct metabolic acidosis / remove lactate

57. If high volume haemofiltration is used on your unit to remove the soluble mediators of sepsis, what dose would you routinely use? (ml/kg/hr)

____

58. What is the maximum CRRT dose used in septic shock on your unit? (ml/kg/hr)

____

AKI AND CRRT

59. What is your unit's modality of choice in infants (<1 year) with acute renal failure, who are haemodynamically stable?

- IHD
- PD
- SLED
- CRRT

60. What is unit's modality of choice in older children (>1year) with acute renal failure, who are hemodynamically stable?

- IHD
- PD
- SLED
- CRRT

61. What is unit's modality of choice in older children (>1year) with acute renal failure, who are hemodynamically stable?

- IHD
- PD
- SLED
- CRRT

62. What factors influence your decision to stop RRT in AKI? (Please rank preferred factors 1-5)

	1	2	3	4	5
Native urine output above a specific threshold					
Calculated native creatinine clearance using serum and urine creatinine					
Resolution of fluid overload					
Clinical improvement					
Logistical factors eg filter has clotted					

63. Is it common practice in your unit to use diuretics (e.g. furosemide) while a child is undergoing RRT

© 2022 Daverio M et al. *JAMA Network Open.*

to augment native urine output?

- Yes
- No

64. On your unit, do nephrologists routinely review patients on RRT?

- Yes
- No

65. Are patients who have received RRT on your unit routinely followed up as outpatients by nephrologists?

- Yes
- No

TRAINING

66. How often do the nursing staff on your unit need to recertify their RRT skills?

- Yearly requirement
- 2 yearly requirement
- Other (please specify) __________

67. Which nursing staff on your unit are trained in the use of RRT?

- All permanent ICU nursing staff trained
- Only selected staff are trained

68. Does your unit offer CRRT training courses for doctors and nurses?

- No regular courses offered
- Yearly
- Six monthly
- More regularly than six months

69. Does your unit organise CRRT simulation training for staff?

- Yes
- No

70. About fluid removal: I begin removing fluid in the first hour of CKRT:

- Always
- Sometimes
- Never

71. My primary consideration when deciding to initiate net ultrafiltration is:

- Fluid Balance
- Haemodynamic status
- Combination of the 2
- Team discretion

72. Do you have a maximum net ultrafiltration rate in mls/kg/hour?

- Yes
- No

73. If Yes to the above question, what is the maximum net ultrafiltrationrate in mls/kg/hour?

____

74. I base my maximum fluid removal goal on:

© 2022 Daverio M et al. *JAMA Network Open.*

- Patient’s hemodynamic status
- Patient’s total body volume status
- Combination of the 2
- Team discretion

75. I assess fluid removal goals at least every:

- 4 hours
- 6 hours
- 12 hours
- 24 hours
- Variable

76. I monitor filtration fraction

- Always
- Sometimes
- Never

77. Whilst stopping CKRT what is your preference

- Fluid bolus followed by diuretic
- Diuretic bolus
- Diuretic bolus followed by infusion
- None of the above, wait for spontaneous diuresis
- Variable

78. Whilst dosing CRRT, what do you use:

- Weight based – mls/kg/hour
- Surface area based – mls /1.73 metre square/ hour
- Variable

© 2022 Daverio M et al. *JAMA Network Open.*
