# Supplementary material for: Continuous Kidney Replacement Therapy Practices in Pediatric Intensive Care Units Across Europe
Source: JAMA Netw Open. 2022 Dec 15;5(12):e2246901. doi: 10.1001/jamanetworkopen.2022.46901 (PMC9856326; doi:10.1001/jamanetworkopen.2022.46901)
Supplement: Supplement 2. — Nonauthor Collaborators [file jamanetwopen-e2246901-s002.pdf]

Supplemental Online Content: Nonauthor Collaborators
*First name, last name, and suffix (if applicable) are required and will appear in PubMed.

***Group Name(s): Critical Care Nephrology Section of the European Society of Paediatric and Neonatal Intensive Care**

| ***First Name and Middle Initial(s)** | ***Last Name** | ***Suffix (eg, Jr, III)** | Academic Degrees | Institution | Location (city, state/province, country) | Role or Contribution, eg, chair, principal investigator | Group (if more than 1 Group listed in the byline) and/or Subgroup (eg, Steering Committee) |
|---|---|---|---|---|---|---|---|
| Sue | Taylor | | | King’s College Hospital NHS Foundation Trust | London, UK | Help in collecting data | |
| Emma | Alexander | | | King’s College Hospital NHS Foundation Trust | London, UK | Help in reviewing manuscript | ESPNIC Critical Care Nephrology Section |
| Kate | Peace | | | Nottingham University Hospital NHS Trust | Nottingham, UK | Help in collecting data | |
| Angela | Amigoni | | | PICU - Department of Woman's and Child's Health | Padova, Italy | Help in collecting data | ESPNIC Critical Care Nephrology Section |
| Felix | Neunhoeffer | | | PICU - University Children's Hospital of Tuebingen, Tuebingen, Germany. | Tubingen, Germany | Help in collecting data | |
