# Supplementary material for: Continuous Kidney Replacement Therapy Practices in Pediatric Intensive Care Units Across Europe
Source: JAMA Netw Open. 2022 Dec 15;5(12):e2246901. doi: 10.1001/jamanetworkopen.2022.46901 (PMC9856326; doi:10.1001/jamanetworkopen.2022.46901)
Supplement: Supplement 3. — Data Sharing Statement [file jamanetwopen-e2246901-s003.pdf]

Data Sharing Statement

Daverio. Continuous Kidney Replacement Therapy Practices in Pediatric Intensive Care Units Across Europe. *JAMA Netw Open*. Published December 15, 2022.
doi:10.1001/jamanetworkopen.2022.46901

Data
Data available: Yes
Data types: Data (not involving human participants)
How to access data: marco.daverio.va@gmail.com
When available: With publication

Supporting Documents
Document types: None

Additional Information
Who can access the data: researchers whose proposed use of the data has been approved
Types of analyses: For a specific purpose
Mechanisms of data availability: after approval of a proposal
